# Supplementary material for: Macrophages in epididymal adipose tissue secrete osteopontin to regulate bone homeostasis
Source: Nat Commun. 2022 Jan 20;13:427. doi: 10.1038/s41467-021-27683-w (PMC8776868; doi:10.1038/s41467-021-27683-w)
Supplement: Supplementary file 1 — Supplementary Information [file 41467_2021_27683_MOESM1_ESM.docx]

**Supplementary Information**

**Macrophages in Epididymal Adipose Tissue Secrete Osteopontin to Regulate Bone Homeostasis**

**Dai *et al.***


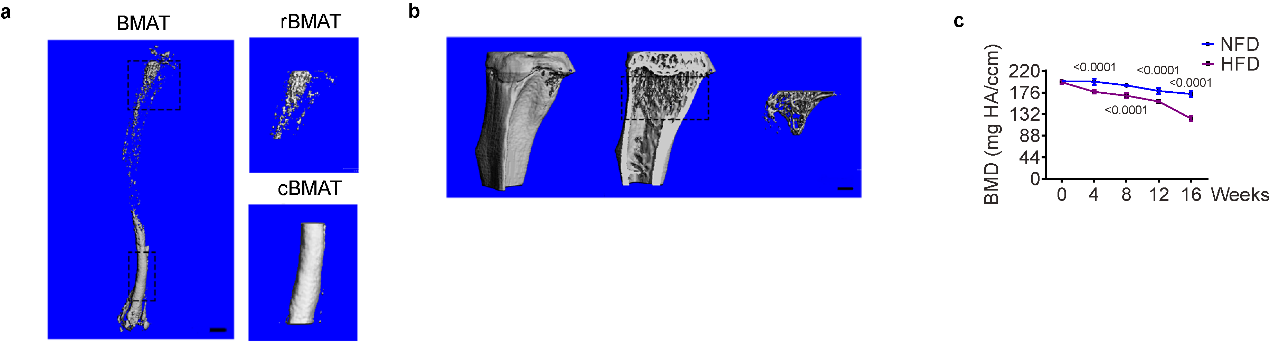


**Supplementary Figure 1. The representative μCT images and quantification of proximal tibiae.**

(a) Regions of interest (ROI) for proximal and distal bone marrow adipose tissue (BMAT) in the tibia (ROI within the dotted frame). Scale bar: 500 μm. (b) ROI for trabecular bone undergrowth plate of the proximal tibia (ROI within the dotted frame). Scale bar: 500 μm. (c) Quantification of bone mineral density (BMD) of proximal tibiae of the NFD- and HFD-fed groups at the indicated time points (*n* = 5 biologically independent samples). Images are representative of 3 independent experiments. All data are presented as mean ± SD. Two-way ANOVA with *Sidak’s post hoc* test (c) was used. Source data are provided as a Source Data file.


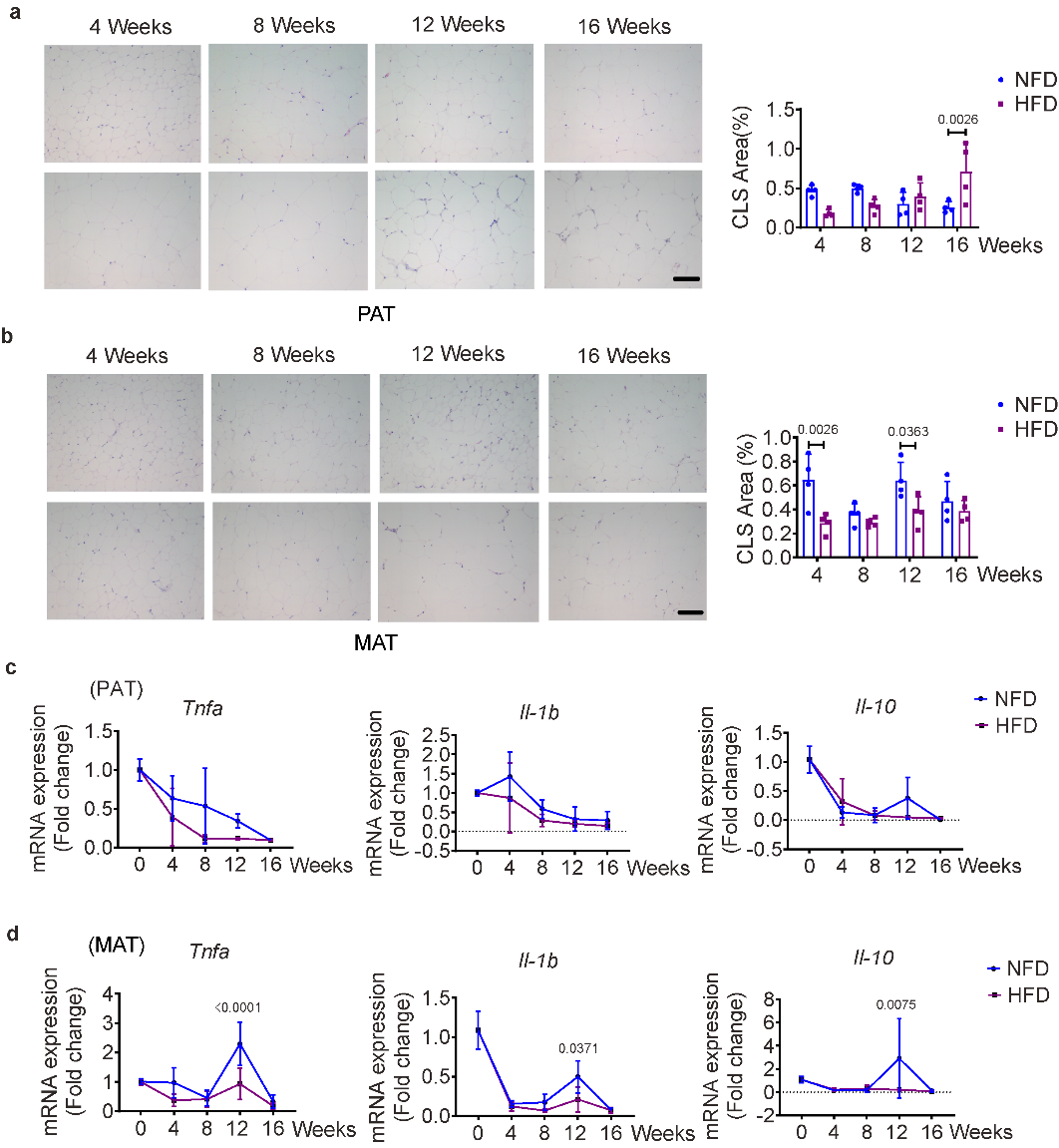


**Supplementary Figure 2. The inflammation in PAT and MAT.**

(a) Representative H&E staining (left) and quantification of CLS area percentage (right, *n* = 4) of PAT from NFD- and HFD-fed mice at the indicated time points. Scale bar: 100 μm. (b) Representative H&E staining (left) and quantification of CLS area percentage (right, *n* = 4) of MAT from NFD- and HFD-fed mice at the indicated time points. Scale bar, 100 μm. (c and d) Relative expression of *Tnfa*, *Il-1b,* and *Il-10* in PAT (*n* = 4) (c) and MAT (*n* = 4) (d), respectively, over the course of the feeding regimens. *n* = 4 biologically independent samples per group. Images are representative of 3 independent experiments. All data are presented as mean ± SD. Two-way ANOVA with *Sidak’s post hoc* test (a, b, c, and d) were used. Source data are provided as a Source Data file.


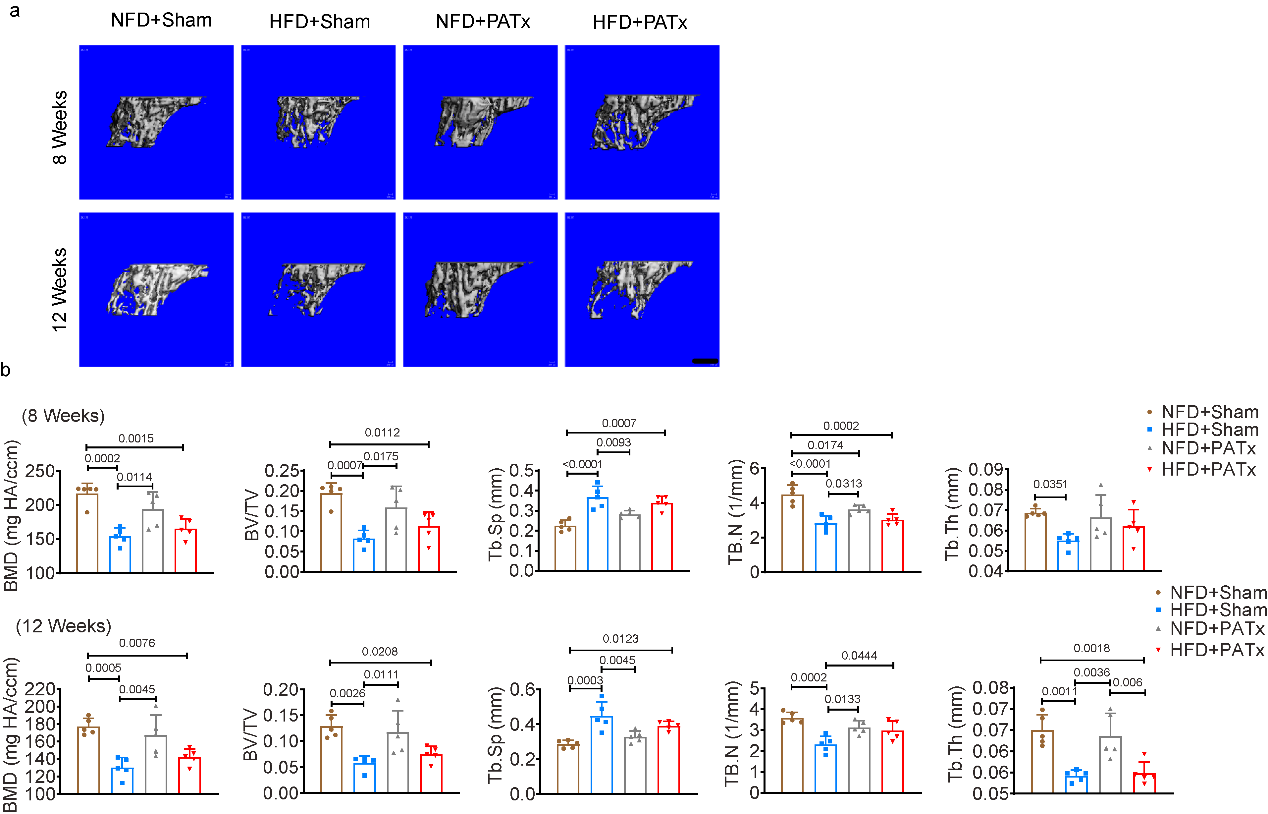


**Supplementary Figure 3. Representative images and quantification of bone microarchitecture at proximal tibiae of NFD- and HFD-fed mice with or without removal of bilateral PATs (PATx).**

(a and b) Representative μCT images (a) and quantification (*n* = 5 biologically independent samples) (b) of proximal tibiae from NFD- and HFD-fed mice that had undergone either sham surgery or removal of bilateral PATs (PATx). Scale bar: 500 μm. Images are representative of 3 independent experiments. All data are presented as mean ± SD. Two-way ANOVA with *Tukey’s post hoc* test (b) were used. Source data are provided as a Source Data file. See also Supplementary Table 9.


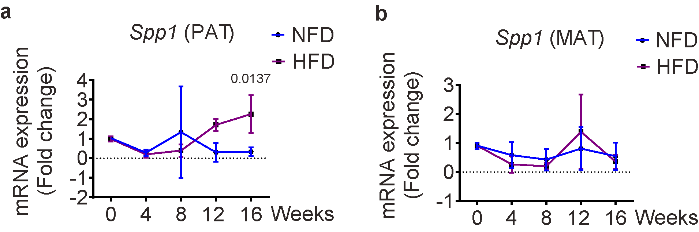


**Supplementary Figure 4. Relative expression of *Spp1* in PAT and MAT.**

Relative expression of *Spp1* was comparable in PAT (a) and MAT (b) over the course of the feeding regimens, except for week 16 in PAT, which was significantly higher in the HFD-fed group as compared with the NFD-fed group (*n* = 4 biologically independent samples). Three independent experiments. All data are presented as mean ± SD. Two-way ANOVA with *Sidak’s post hoc* test (a and b) were used. Source data are provided as a Source Data file.


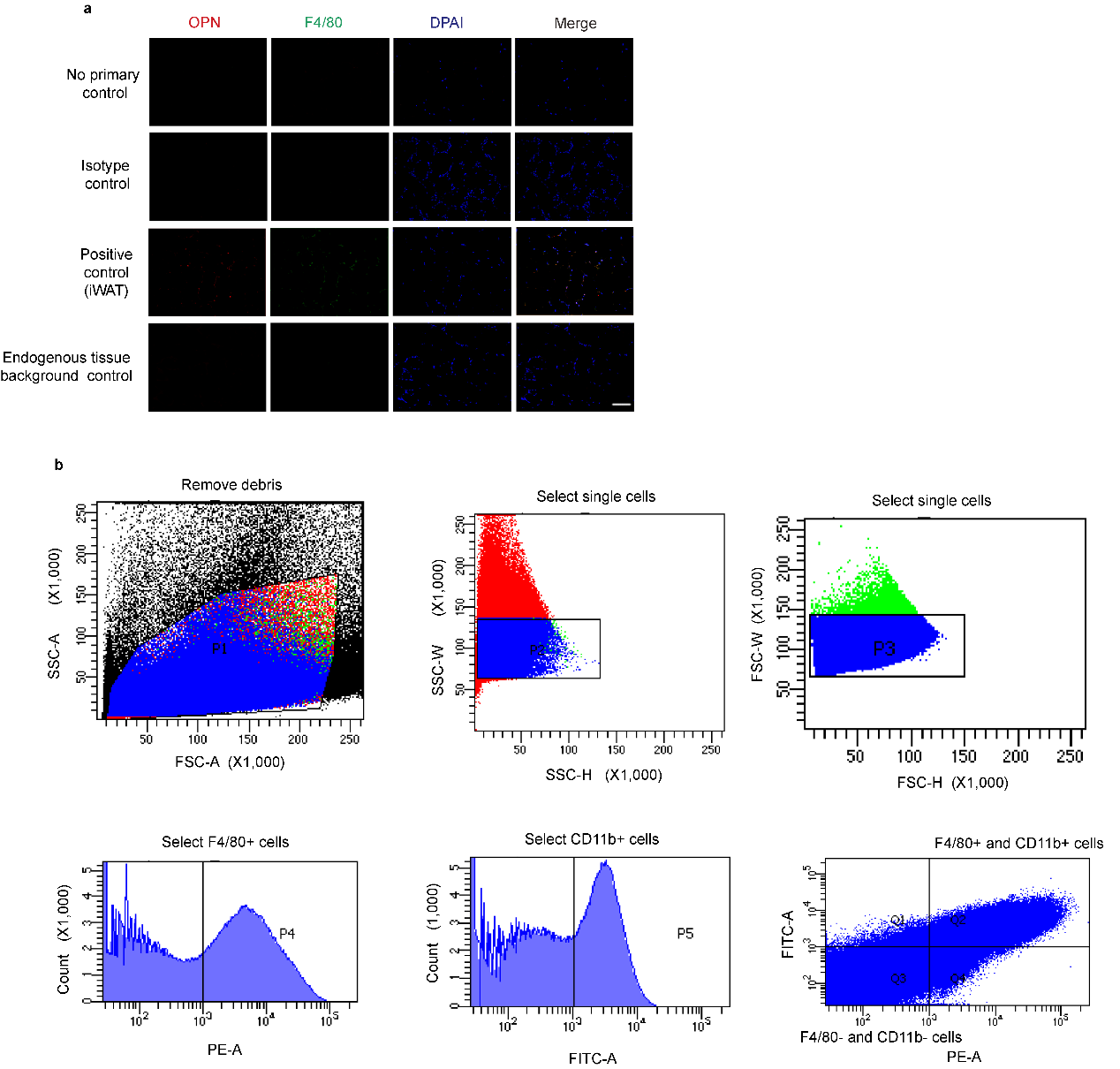


**Supplementary Figure 5. Representative images of control immunofluorescent staining and flow cytometry gating scheme.**

(a) Control immunofluorescent staining for the OPN and F4/80 in eWAT and iWAT (positive control staining). Scale bar: 100 μm. (b) Flow cytometry gating scheme for detecting the percentage of F4/80+ and CD11b+ cells in eWAT. Images are representative of 3 independent experiments.


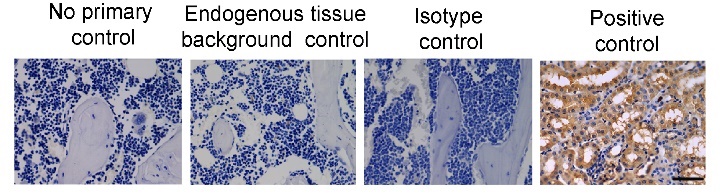


**Supplementary Figure 6. Representative images of control immunohistochemical staining OPN.**

Control immunohistochemical staining of OPN in proximal tibiae and kidney (positive control staining). Scale bar: 50 μm. Images are representative of 3 independent experiments.


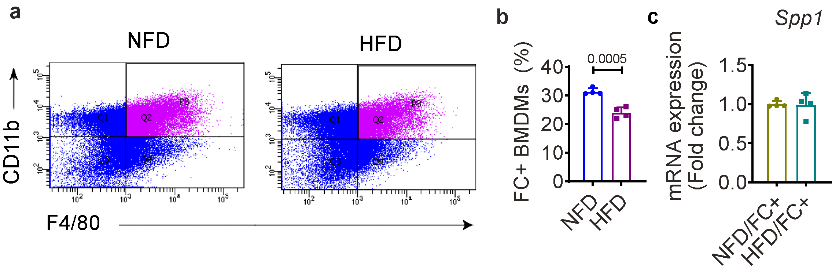


**Supplementary Figure 7. The relative expression of *spp1* in FC+ BMDMs.**

(a) Macrophages gated by FACS for F4/80 and CD11b expression from bone marrow at week 12 of NFD (left) and HFD (right) feeding. Q2 represents the F4/80+ and CD11b+ (FC+) macrophages population. (b) The percentage of FC+ BMDMs from NFD- and HFD-fed mice at week 12 (*n* = 4 biologically independent samples). (c) Relative expression of *Spp1* in FC+ BMDMs from NFD- and HFD-fed mice at week 12 (*n* = 4 biologically independent samples). All data are presented as mean ± SD. Two-tailed *Welch’s t*-test (b and c) were used. Source data are provided as a Source Data file.


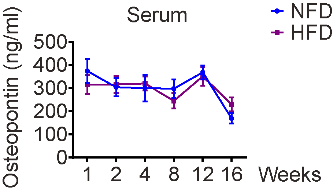


**Supplementary Figure 8. The concentration of OPN in serum.**

OPN level in serum of NFD- and HFD-fed groups at weeks 1, 2, 4, 8, 12, and 16 (*n* = 5 biologically independent samples). Three independent experiments. All data are presented as mean ± SD. Two-way ANOVA with *Sidak’s post hoc* test was used. Source data are provided as a Source Data file.


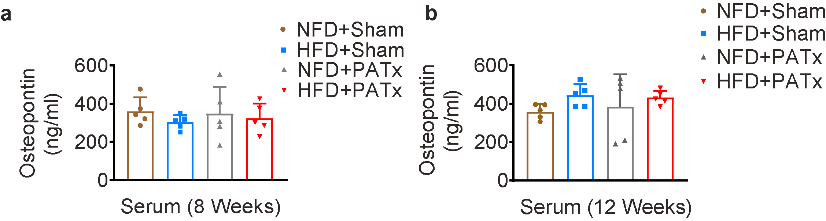


**Supplementary Figure 9. OPN expression in serum of NFD- and HFD-fed groups with or without PATx.**

(a, b) OPN expression in serum of NFD- and HFD-fed groups with or without PATx at weeks 8 and 12 (*n* = 5 biologically independent samples). Three independent experiments. All data are presented as mean ± SD. Two-way ANOVA with *Tukey’s post hoc* test (a and b) were used. Source data are provided as a Source Data file. See also Supplementary Table 10 and 11.


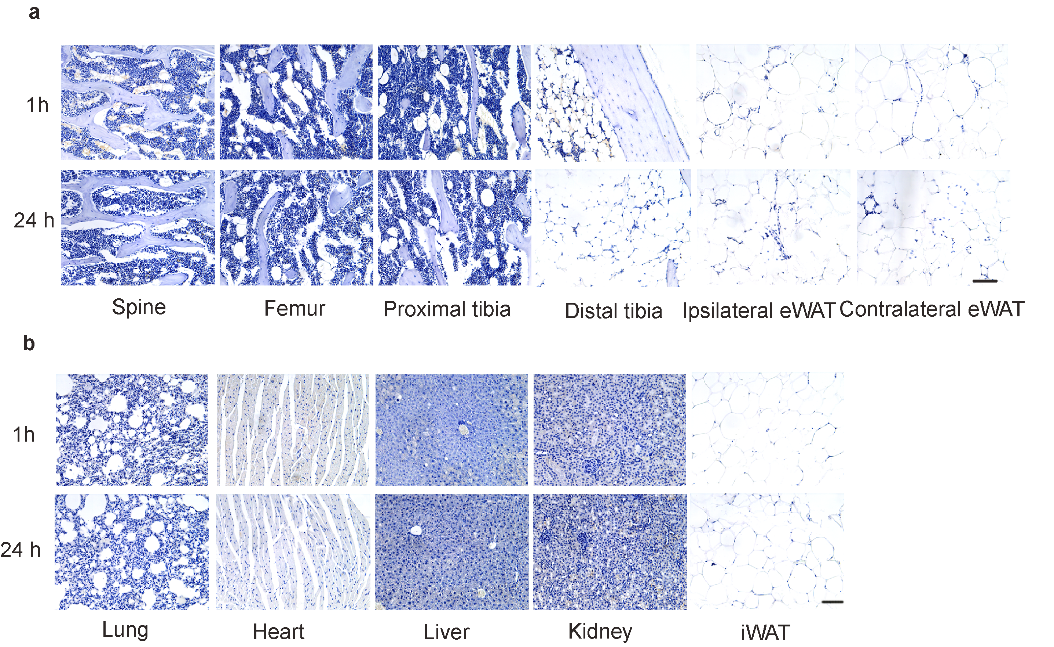


**Supplementary Figure 10. Representative images of immunohistochemical staining of OPN.**

(a and b) Representative images of immunohistochemical staining of OPN (anti-human OPN antibody) in spine, femur, tibia (proximal and distal), eWAT (ipsilaterally-injected depot and contralateral depot) (a), lung, heart, liver, kidney, and iWAT (b) of mice unilaterally injected with saline into the eWAT for 1 hour (top row) and 24 hours (bottom row), respectively. Scale bar: 100 μm. Images are representative of 3 independent experiments.


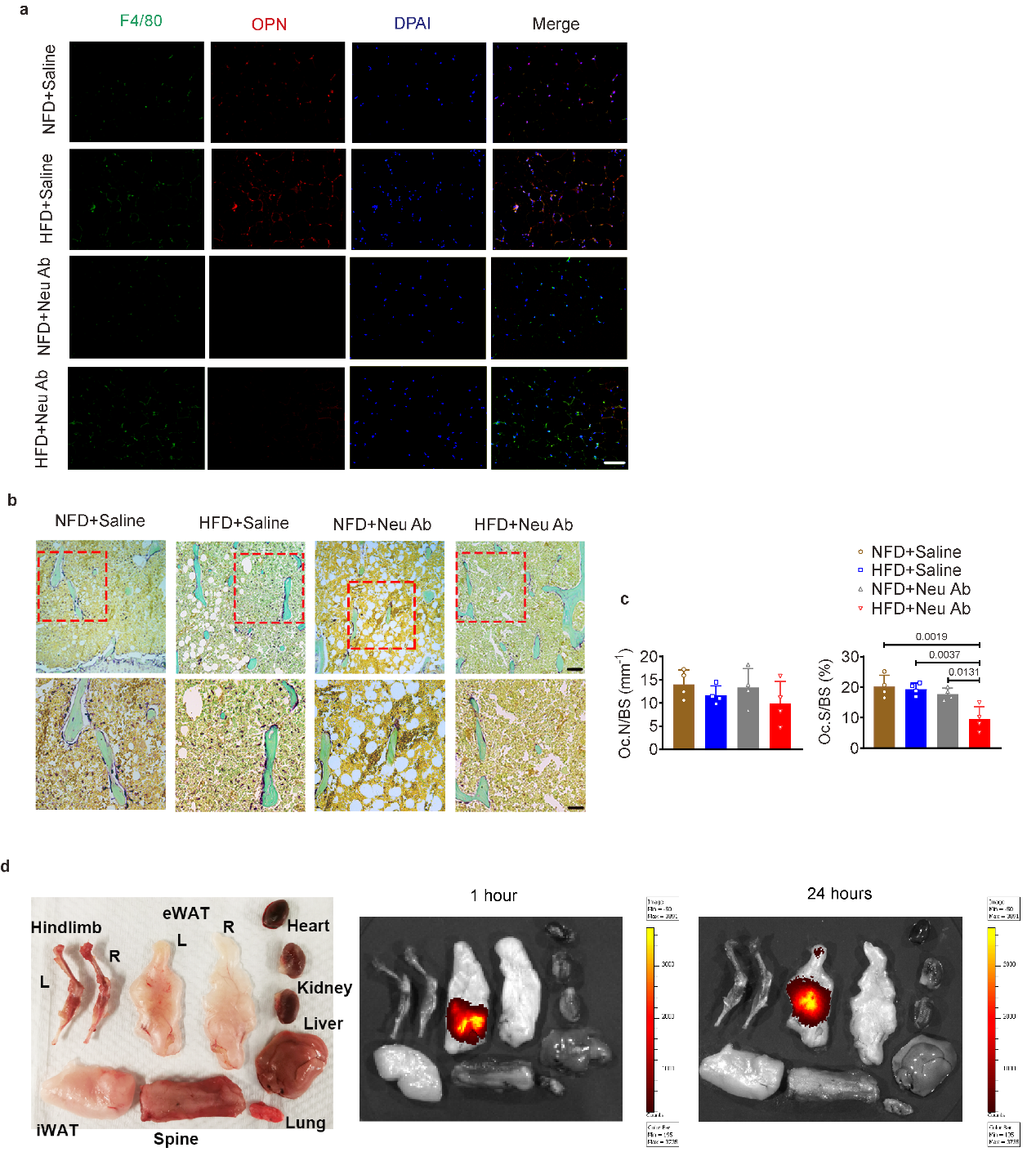


**Supplementary Figure 11. The alterations of eWAT and proximal tibiae in HFD-fed mice after injection of saline or OPN neutralizing antibody into bilateral eWATs for 8 weeks.**

(a) Immunofluorescent staining of ATMs (F4/80) and OPN in eWAT after injection of OPN neutralizing antibody (Neu Ab) or saline into bilateral eWATs for 8 weeks. Red, OPN; green, F4/80; blue, DAPI stain for cell nuclei. Scale bar: 75 μm. (b and c) Representative images of TRAP staining (Bottom row is a magnification of the top row. Scale bar: 50 µm) (b) and quantification of Trap+ osteoclast number and surface (*n* = 4 biologically independent samples) (c) in the proximal tibiae after mice injected saline or Neu Ab into bilateral eWATs for 8 weeks. Scale bar: 100 μm. Oc.N/BS, osteoclast number per bone surface; Oc.S/BS, osteoclast surface per bone surface. (d) *Ex vivo* imaging (IVIS200 system) of the indicated organs and tissues collected at 1 hour (left part in the right box) and 24 hours (right part in the right box) after unilateral injection of OPN neutralizing antibody conjugated with FITC (Neu Ab-FITC) into the eWAT. Images are representative of 3 independent experiments. All data are presented as mean ± SD. Two-way ANOVA with *Tukey’s* *post hoc* test (c) were used. Source data are provided as a Source Data file. See also Supplementary Table 12.


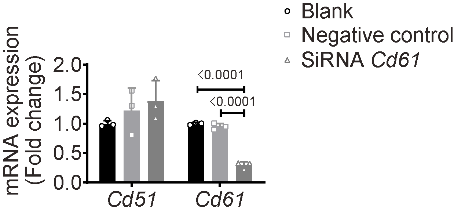


**Supplementary Figure 12. The transfection efficiency of small interfering RNA *Cd61*** **(SiRNA *integrin β3*).**

The transfection efficiency of SiRNA *Cd61* at day 1 post-transfection (*n* = 3 biologically independent samples). Three independent experiments. All data are presented as mean ± SD. One-way ANOVA with *Tukey’s post hoc* test was used. Source data are provided as a Source Data file.


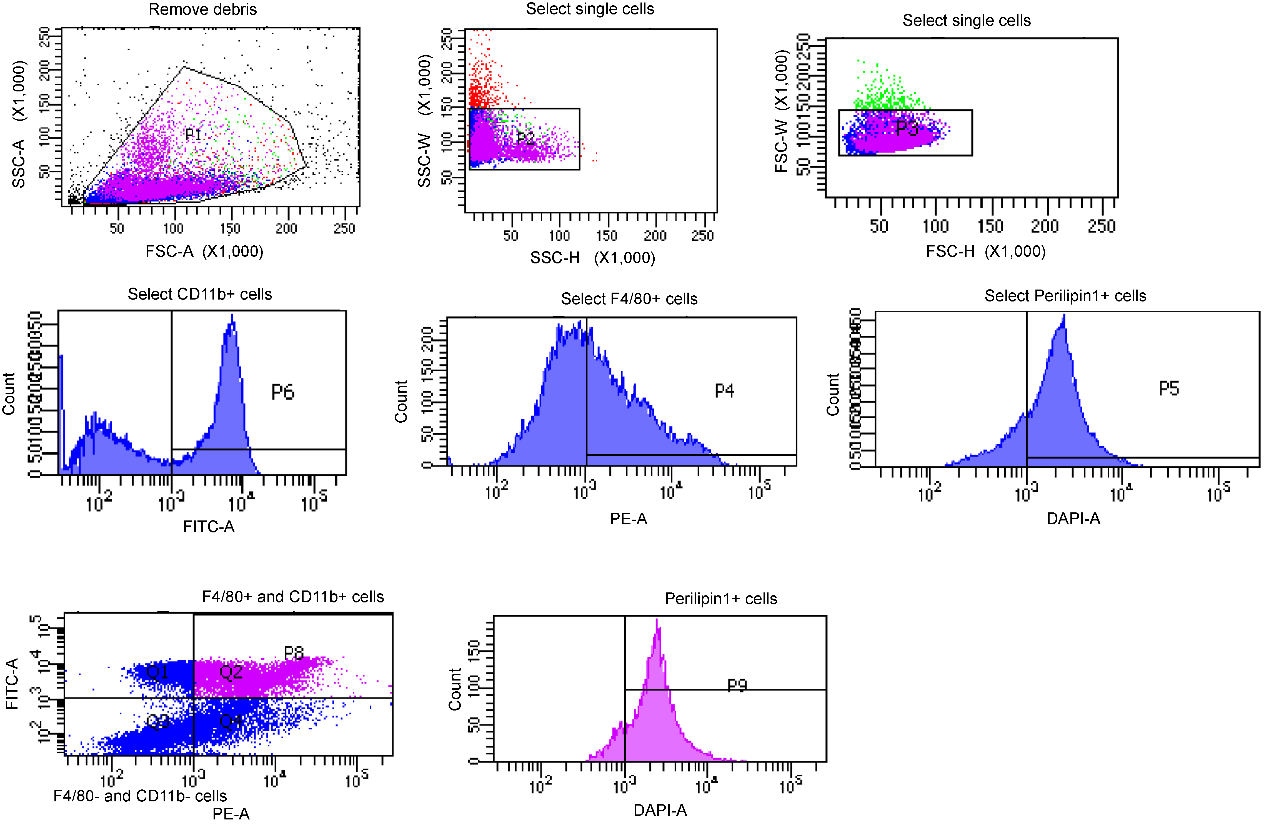


**Supplementary Figure 13. Representative images of flow cytometry gating scheme.**

Flow cytometry gating scheme for detecting the percentage of FC+ and Perilipin1+ cells in BMDMs.


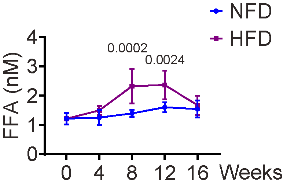


**Supplementary Figure 14. Free fatty acid (FFA) level in the bone marrow supernatant of tibiae.**

FFA level in the bone marrow supernatant of tibiae (*n* = 5 biologically independent samples). Three independent experiments. All data are presented as mean ± SD. Two-way ANOVA with *Sidak’s post hoc* test was used. Source data are provided as a Source Data file.


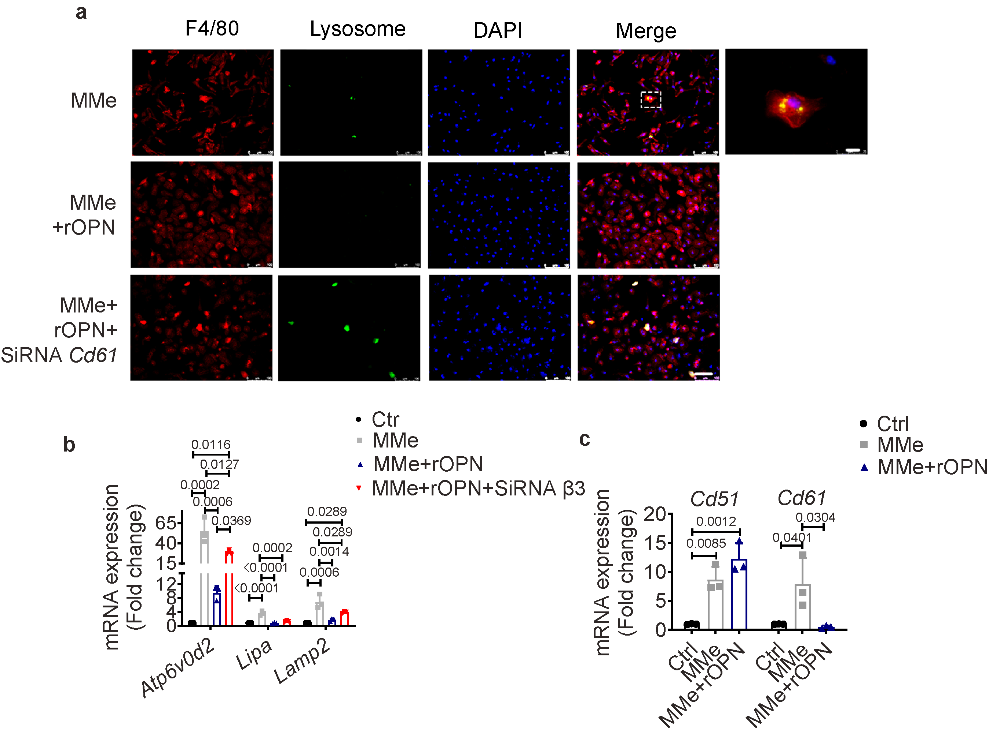


**Supplementary Figure 15. Immunofluorescent images of metabolic activation of macrophage (MMe) and relative expression of *Cd51* and *Cd61*** (***integrin αv* and *integrin β3*) in MMe.**

(a) Immunofluorescent images of MMe stained for lysosome and macrophage (F4/80) after treatment with rOPN or SiRNA *Cd61* for 24 hours as indicated *in vitro*. Scale bar: 50 μm. The right most picture is an enlarged view of the field in the white dashed box. Scale bar: 12.5 μm. (b) Relative expression of *Atp6v0d2*, *Lamp2*, and *Lipa* in BMDM (ctrl) and MMe treated with rOPN or SiRNA *Cd61* for 24 hours as indicated (*n* = 3 biologically independent samples). (c) Relative expression of *Cd51* and *Cd61* in BMDM (ctrl), MMe, and MMe treated with rOPN (0.5 μg/ml) for 24 hours *in vitro* (*n* = 3 biologically independent samples). Images are representative of 3 independent experiments. All data are presented as mean ± SD. One-way ANOVA with *Tukey’s post hoc* test (b and c) were used. Source data are provided as a Source Data file.

**Supplementary Table 1. Primer Sequences used for qRT-PCR.**

| Gene name | Oligonucleotide primers |
| --- | --- |
| *Atp6v0a2*-F | TGGTGCAGTTCCGAGACCT |
| *Atp6v0a2*-R | GCAGGGGAATATCAGCTCTGG |
| *Atp6v0c*-F | TGTCCCGTTGTCCTAGCTC |
| *Atp6v0c*-R | CCATGACACCGAAAAACGAAGA |
| *Atp6v0d1*-F | GCTACTTGGAGGGATTAGTGCG |
| *Atp6v0d1*-R | GCGGAACTCTACTACCATCTTCT |
| *Atp6v1a*-F | CTACCCAAAATCCGCGATGAG |
| *Atp6v1a*-R | CCATGTCACCTTCCAATCGAA |
| *Atp6v1c1*-F | ACTGAGTTCTGGCTCATATCTGC |
| *Atp6v1c1*-R | TGGAAGAGACGGCAAGATTATTG |
| *Atp6v1d*-F | GGCAAAGACCGGATTGAAATCT |
| *Atp6v1d*-R | GTCGAAATCGAAGAGTTAAGGCA |
| *Atp6v0a3*-F | CACAGGGTCTGCTTACAACTG |
| *Atp6v0a3*-R | CGTCTACCACGAAGCGTCTC |
| *Atp6v1g1*-F | CCCAGGCTGAAATTGAACAGT |
| *Atp6v1g1*-R | TTCTGGAGGACGGTCATCTTC |
| *Atp6v0d2*-F | AGCCAGCCTAACTCAGC |
| *Atp6v0d2*-R | GCTTCTTCCTCATCTCCGTGTC |
| *Spp1*-F | AGCAAGAAACTCTTCCAAGCAA |
| *Spp1*-R | GTGAGATTCGTCAGATTCATCCG |
| *Lamp2*-F | ATGTGCCTCTCTCCGGTTAAA |
| *Lamp2*-R | GCAAGTACCCTTTGAATCTGTCA |
| *Lipa*-F | TGTTCGTTTTCACCATTGGGA |
| *Lipa*-R | CGCATGATTATCTCGGTCACA |
| *Il-1b*-F | TTCAGGCAGGCAGTATCACTC |
| *Il-1b*-R | GAAGGTCCACGGGAAAGACAC |
| *Il-10*-F | GCTCTTACTGACTGGCATGAG |
| *Il-10*-R | CGCAGCTCTAGGAGCATGTG |
| *Tnfa*-F | CCTGTAGCCCACGTCGTAG |
| *Tnfa*-R | GGGAGTAGACAAGGTACAACCC |
| *Mmp9*-F | CTGGACAGCCAGACACTAAAG |
| *Mmp9*-R | CTCGCGGCAAGTCTTCAGAG |
| *Cd51*-F | CCGTGGACTTCTTCGAGCC |
| *Cd51*-R | CTGTTGAATCAAACTCAATGGGC |
| *Cd61*-F | CCACACGAGGCGTGAACTC |
| *Cd61*-R | CTTCAGGTTACATCGGGGTGA |

**Supplementary Table 2. The *p* values of the main and interaction effects (Related to Fig. 3b).**

8 weeks

|  | eWATx effect (*p* value) | Interaction effect (*p* value) |
| --- | --- | --- |
| BMD | 0.0339 | 0.1468 |
| BV/TV | 0.0199 | 0.4008 |
| Tb.Sp | 0.5931 | 0.1302 |
| Tb.N | 0.3684 | 0.4978 |
| Tb.Th | 0.0004 | 0.0216 |

12 weeks

|  | eWATx effect (*p* value) | Interaction effect (*p* value) |
| --- | --- | --- |
| BMD | 0.0003 | 0.0668 |
| BV/TV | 0.0053 | 0.1185 |
| Tb.Sp | 0.0003 | 0.8166 |
| Tb.N | 0.0686 | 0.0778 |
| Tb.Th | 0.0055 | 0.1263 |

**Supplementary Table 3. The *p* values of the main and interaction effects (Related to Fig. 3d).**

|  | eWATx effect (*p* value) | Interaction effect (*p* value) |
| --- | --- | --- |
|  | 0.0149 | 0.0141 |

**Supplementary Table 4. The *p* values of the main and interaction effects (Related to Fig. 4d).**

|  | CL injection effect (*p* value) | Interaction effect (*p* value) |
| --- | --- | --- |
| BMD | <0.0001 | 0.0656 |
| BV/TV | 0.0016 | 0.0926 |
| Tb.Sp | 0.0001 | 0.2530 |
| Tb.N | 0.0001 | 0.3359 |
| Tb.Th | 0.0264 | 0.1685 |

**Supplementary Table 5. The *p* values of the main and interaction effects (Related to Fig. 4e).**

|  | CL injection effect (*p* value) | Interaction effect (*p* value) |
| --- | --- | --- |
| rBMAT mass (%) | 0.0162 | 0.0323 |

**Supplementary Table 6. The *p* values of the main and interaction effects (Related to Fig. 6d).**

|  | eWATx effect (*p* value) | Interaction effect (*p* value) |
| --- | --- | --- |
| Osteopontin (ng/ml) | 0.0001 | 0.8615 |

**Supplementary Table 7. The *p* values of the main and interaction effects (Related to Fig. 7j).**

|  | Neu Ab injection effect (*p* value) | Interaction effect (*p* value) |
| --- | --- | --- |
| BMD | 0.1111 | 0.019 |
| BV/TV | 0.1393 | 0.0147 |
| Tb.Sp | 0.8933 | 0.9055 |
| Tb.N | 0.8296 | 0.9238 |
| Tb.Th | 0.2112 | 0.0126 |

**Supplementary Table 8. The *p* values of the main and interaction effects (Related to Fig. 7l).**

|  | Neu Ab injection effect (*p* value) | Interaction effect (*p* value) |
| --- | --- | --- |
| rBMAT mass (%) | 0.0213 | 0.0207 |

**Supplementary Table 9. The *p* values of the main and interaction effects (Related to Supplementary Fig. 3b).**

8 weeks

|  | PATx effect (*p* value) | Interaction effect (*p* value) |
| --- | --- | --- |
| BMD | 0.4852 | 0.0464 |
| BV/TV | 0.8788 | 0.0548 |
| Tb.Sp | 0.3818 | 0.0185 |
| Tb.N | 0.0846 | 0.0091 |
| Tb.Th | 0.4557 | 0.1620 |

12 weeks

|  | PATx effect (*p* value) | Interaction effect (*p* value) |
| --- | --- | --- |
| BMD | 0.8555 | 0.1039 |
| BV/TV | 0.8269 | 0.2341 |
| Tb.Sp | 0.7324 | 0.0321 |
| Tb.N | 0.5261 | 0.0031 |
| Tb.Th | 0.8142 | 0.5547 |

**Supplementary Table 10. The *p* values of the main and interaction effects (Related to Supplementary Fig. 9a).**

|  | PATx effect (*p* value) | Interaction effect (*p* value) |
| --- | --- | --- |
| Osteopontin (ng/ml) | 0.9224 | 0.6894 |

**Supplementary Table 11. The *p* values of the main and interaction effects (Related to Supplementary Fig. 9b).**

|  | PATx effect (*p* value) | Interaction effect (*p* value) |
| --- | --- | --- |
| Osteopontin (ng/ml) | 0.8841 | 0.6592 |

**Supplementary Table 12. The *p* values of the main and interaction effects (Related to Supplementary Fig. 11c).**

|  | PATx effect (*p* value) | Interaction effect (*p* value) |
| --- | --- | --- |
| Oc.N/BS (mm^-1^) | 0.5128 | 0.7837 |
| Oc.S/BS (%) | 0.0019 | 0.0356 |
