# Supplementary material for: Macrophages in epididymal adipose tissue secrete osteopontin to regulate bone homeostasis
Source: Nat Commun. 2022 Jan 20;13:427. doi: 10.1038/s41467-021-27683-w (PMC8776868; doi:10.1038/s41467-021-27683-w)
Supplement: Supplementary file 3 — Reporting Summary [file 41467_2021_27683_MOESM3_ESM.pdf]

nature portfolio

Corresponding author(s): Ling Qin

Last updated by author(s): Aug 10, 2021

Reporting Summary

Nature Portfolio wishes to improve the reproducibility of the work that we publish. This form provides structure for consistency and transparency in reporting. For further information on Nature Portfolio policies, see our Editorial Policies and the Editorial Policy Checklist.

Statistics

For all statistical analyses, confirm that the following items are present in the figure legend, table legend, main text, or Methods section.

n/a	Confirmed	
☐	☒	The exact sample size (*n*) for each experimental group/condition, given as a discrete number and unit of measurement
☐	☒	A statement on whether measurements were taken from distinct samples or whether the same sample was measured repeatedly
☐	☒	The statistical test(s) used AND whether they are one- or two-sided<br>*Only common tests should be described solely by name; describe more complex techniques in the Methods section.*
☐	☒	A description of all covariates tested
☐	☒	A description of any assumptions or corrections, such as tests of normality and adjustment for multiple comparisons
☐	☒	A full description of the statistical parameters including central tendency (e.g. means) or other basic estimates (e.g. regression coefficient) AND variation (e.g. standard deviation) or associated estimates of uncertainty (e.g. confidence intervals)
☐	☒	For null hypothesis testing, the test statistic (e.g. *F*, *t*, *r*) with confidence intervals, effect sizes, degrees of freedom and *P* value noted<br>*Give P values as exact values whenever suitable.*
☒	☐	For Bayesian analysis, information on the choice of priors and Markov chain Monte Carlo settings
☒	☐	For hierarchical and complex designs, identification of the appropriate level for tests and full reporting of outcomes
☒	☐	Estimates of effect sizes (e.g. Cohen's *d*, Pearson's *r*), indicating how they were calculated

Our web collection on statistics for biologists contains articles on many of the points above.

Software and code

Policy information about availability of computer code

Data collection	Images were acquired using Leica DM5500; Leica Micro-systems. Western blot data was acquired digitally by GeneGnome XRQ. qPCR data was acquired using the Quantstudio TM 12K Flex Real-time PCR. MicorCT scanning was conducted by µCT-40 (Scanco Medical, Brüttisellen, Switzerland). In vivo imaging of different organs in a mouse after injection of FITC-labeled recombinant human-OPN or FITC-labeled OPN neutralizing antibody by an IVIS200 imaging system, Xenogen Imaging Technologies. Pits were observed as bright spots on the slides using a microscope (ZEISS AxioPlan2, Germany). Migrated BMDMs in transwell assay were observed using a microscope (ZEISS AxioPlan2, Germany). RNA was quantified spectrophotometrically based on the A260 using a ND-2000 spectrophotometer (NanoDrop Technologies, Wilmington, DE, USA).
Data analysis	Data were analyzed using Prism Graphpad version 8.2.1. Microscopy images analysis was performed using ImageJ version 1.52v. Densitometric analysis of western blots were performed by Gene Sys V1.6.1.0 and ImageJ version 1.52v. qPCR data analysis was acquired using the Quantstudio TM 12K Flex Software v 1.3. RNA was quantified using a ND-2000 version 1.5. Images of IVIS200 imaging system were acquired by Living image version 3.2. Heatmap was created by Heatmap Illustrator (version Heml 1.0.3.7). Data of FACS were analyzed using BD FACSDiva software v8.0.1.

For manuscripts utilizing custom algorithms or software that are central to the research but not yet described in published literature, software must be made available to editors and reviewers. We strongly encourage code deposition in a community repository (e.g. GitHub). See the Nature Portfolio guidelines for submitting code & software for further information.

Data

Policy information about availability of data

All manuscripts must include a data availability statement. This statement should provide the following information, where applicable:

- Accession codes, unique identifiers, or web links for publicly available datasets
- A description of any restrictions on data availability
- For clinical datasets or third party data, please ensure that the statement adheres to our policy

The authors declare that all data supporting the findings of this study are available within the paper and its Supplementary Information files, and from the authors on request. Microarray data files were acquired from the Gene Expression Omnibus with the accession number GSE27017 (this data was used to re-confirm our results).

Field-specific reporting

Please select the one below that is the best fit for your research. If you are not sure, read the appropriate sections before making your selection.

[x] Life sciences [ ] Behavioural & social sciences [ ] Ecological, evolutionary & environmental sciences

For a reference copy of the document with all sections, see nature.com/documents/nr-reporting-summary-flat.pdf

Life sciences study design

All studies must disclose on these points even when the disclosure is negative.

Sample size	To estimate the minimum sample size for both in vitro and in vivo experiments, we calculated the population mean based on our preliminary results by the formula (Response Fig. 1).
Data exclusions	Exclusion criteria for animals were applied in case of death, cannibalism, and severe infection. Exclusion criteria for samples were applied in case of histological artifacts (damaged tissues), RNA and protein degradation.
Replication	All of experiments were successfully repeated at least three times and/or with sufficient cells/animals per group to demonstrate statistical significance. All experiments were statistically analyzed.
Randomization	All the mice were randomly assigned to the indicated groups.
Blinding	The performances were not blinded to the distributions, treatments, and assessments, but the results were confirmed by three independent investigators.

Reporting for specific materials, systems and methods

We require information from authors about some types of materials, experimental systems and methods used in many studies. Here, indicate whether each material, system or method listed is relevant to your study. If you are not sure if a list item applies to your research, read the appropriate section before selecting a response.

Materials & experimental systems

n/a	Involved in the study	
[ ]	[x]	Antibodies
[x]	[ ]	Eukaryotic cell lines
[x]	[ ]	Palaeontology and archaeology
[ ]	[x]	Animals and other organisms
[x]	[ ]	Human research participants
[x]	[ ]	Clinical data
[x]	[ ]	Dual use research of concern

Methods

n/a	Involved in the study	
[x]	[ ]	ChIP-seq
[ ]	[x]	Flow cytometry
[x]	[ ]	MRI-based neuroimaging

Antibodies

Antibodies used	Cathepsin K (Abcam, ab37259), Osteopontin (Thermo Fisher Scientific, PA125152), Osteopontin (Abcam, ab8448), Osteopontin (Abcam, ab92964), Osteopontin (Abcam, ab214050), F4/80 (Thermo Fisher Scientific, 12-4801-80), F4/80 (eBioscience, 11-4801-85), CD11b (Thermo Fisher Scientific, 53-0112-80), Lysotracker (Thermo Fisher Scientific, L7526), Donkey anti-rabbit 405 (Abcam, ab175651), Goat anti-rabbit 488 (Abcam, ab150077), Donkey anti-rabbit 594 (Abcam, ab150076), Donkey anti-Goat 488 (Abcam, ab150129), Donkey anti-mouse 405 (Abcam, ab175658), Integrin b3 (Abcam, ab38460), Integrin av (Abcam, ab179475), Perilipin 1 (Abcam, ab3526), MMP 9 (Abcam, ab38898), JNK (Cell Signaling Technology, 9252s), P-JNK (Cell Signaling Technology,9255s), DAPI

	(Invitrogen, P36935), ATP6V0d2 (Bioss, bs-12548R), Rat IgG2a kappa isotype control (Thermo Fisher Scientific, 13-4321-81, Recombinant rabbit IgG isotype (Abcam, ab172730), β-actin (Cell Signaling Technology, 4970s). Antibody dilution factor mentioned in the method section.
Validation	Antibodies were chosen based on previous literature. Validation and quality control is available from the manufacturers using the catalog number of each antibody.<br>Cathepsin K (ab37259, Dai et al,. Bioact Mater. 2021 Feb 3;6(8):2511-2522. doi: 10.1016/j.bioactmat.2021.01.027.),<br>Osteopontin (PA125152, Yang et al,. J Neuroinflammation. 2019 Aug 19;16(1):169. doi: 10.1186/s12974-019-1527-z.),<br>Osteopontin (ab8448, Lin et al,. Aging Cell. 2020 Mar;19(3):e13091. doi: 10.1111/acel.13091),<br>Osteopontin (ab92964, Tan et al,. J Clin Invest. 2021 Jan 4;131(1):e137186. doi: 10.1172/JCI137186),<br>Osteopontin (ab214050, Zhu et al,. J Biol Chem. 2019 Dec 20;294(51):19465-19474. doi: 10.1074/jbc.RA119.009758.),<br>F4/80 (12-4801-80, Kersten et al,. Oncoimmunology. 2017 Jun 19;6(8):e1334744.),<br>F4/80 (11-4801-85, Jiang et al,. Oncoimmunology. 2016 Aug 3;5(9):e1211219. doi: 10.1080/2162402X.2016.1211219.),<br>CD11b (53-0112-80, Andersohn, et al,. Front Cell Dev Biol. 2019 Sep 18;7:198. doi: 10.3389/fcell.2019.00198.),<br>Lysotracker (L7526, Li et al,. Biology (Basel). 2020 Jan 7;9(1):13. doi: 10.3390/biology9010013.),<br>Donkey anti-rabbit 405 (ab175651, Clawson et al,. Nat Commun. 2021 Feb 22;12(1):1200. doi: 10.1038/s41467-021-21471-2.),<br>Goat anti-rabbit 488 (ab150077, Ishikawa et al,. Autophagy. 2021 Mar;17(3):743-760. doi: 10.1080/15548627.2020.1731270.),<br>Donkey anti-rabbit 594 (ab150076, Hoseini et al,. Elife. 2021 Apr 12;10:e61437. doi: 10.7554/eLife.61437.),<br>Donkey anti-Goat 488 (ab150129, Wang et al,. Front Immunol. 2021 Mar 11;12:641999. doi: 10.3389/fimmu.2021.641999.),<br>Donkey anti-mouse 405 (ab175658, Oscos et al,. Sci Adv . 2021 Feb 3;7(6):eabd2827. doi: 10.1126/sciadv.abd2827.),<br>Integrin b3 (ab38460, Naik et al,. Blood. 2012 Apr 5;119(14):3352-60. doi: 10.1182/blood-2011-12-397398.),<br>Integrin av (ab179475, Jiang et al,. Nat Commun. 22020 Nov 6;11(1):5653. doi: 10.1038/s41467-020-19425-1.),<br>Perilipin 1 (ab3526, Mori et al,. PLoS Biol. 2021 May 12;19(5):e3000988. doi: 10.1371/journal.pbio.3000988.),<br>MMP 9 (ab38898, Zhou et al,. FEBS Open Bio. 2021 May;11(5):1487-1496. doi: 10.1002/2211-5463.12768.),<br>JNK (9252s, Chen et al,. Front Pharmacol. 2021 Jun 3;12:664836. doi: 10.3389/fphar.2021.664836.),<br>P-JNK (9255s, Deng et al,. J Cancer 2021; 12(15):4710-4721. doi:10.7150/jca.58873),<br>DAPI (P36935, Zeng et al,. Nat Methods. 2009 Mar;6(3):207-9. doi: 10.1038/nmeth.1305. Epub 2009 Feb 22.),<br>ATP6V0d2 (Bioss, bs-12548R, patterns were validated in our hands using "no-primary" controls.),<br>Rat IgG2a kappa isotype control (13-4321-81, Böttcher et al,. J Cell Biol. 2017 Nov 6;216(11):3785-3798. doi: 10.1083/jcb.201701176.),<br>Recombinant rabbit IgG isotype (ab172730, Radhakrishnan et al,. Diabetes. 2021 Jan;70(1):227-239. doi: 10.2337/db20-0375. Epub 2020 Oct 13.),<br>β-actin (4970s, Chen et al,. Nat Med. 2009 Nov;15(11):1307-11. doi: 10.1038/nm.2049).<br>Antibody dilution factor mentioned in the method section.

Animals and other organisms

Policy information about studies involving animals; ARRIVE guidelines recommended for reporting animal research

Laboratory animals	Stated in Materials and Methods under "Animals": Twelve-week-old male C57BL/6 mice were fed with a high-fat diet (HFD; 60% of total calories from fat, 20% from carbohydrate and 20% from protein, cat# D12492, Research Diets Inc.). The control group received a normal-fat diet (NFD; 10% of total calories from fat, 70% from carbohydrate and 20% from protein, cat# D12450J,Research Diets Inc.). All animals were housed under a 12-hour light/dark cycle, ambient temperature of 18-23 degree celsius, and 70% humidity at the Experimental Animal Center at the Prince of Wales Hospital in Hong Kong and received NFD/HFD and water ad libitum.
Wild animals	The study did not involved in wild animals.
Field-collected samples	The study did not involved in samples collected from the field.
Ethics oversight	The specified experimental protocols were approved by the Animal Experiment Ethics Committee of the Chinese University of Hong Kong (17-184-MIS-5-C, 19-036-MIS-5-C, 20-111-MIS-5-B, and 21-027-MIS).

Note that full information on the approval of the study protocol must also be provided in the manuscript.

Flow Cytometry

Plots

Confirm that:

- [x] The axis labels state the marker and fluorochrome used (e.g. CD4-FITC).
- [x] The axis scales are clearly visible. Include numbers along axes only for bottom left plot of group (a 'group' is an analysis of identical markers).
- [x] All plots are contour plots with outliers or pseudocolor plots.
- [x] A numerical value for number of cells or percentage (with statistics) is provided.

Methodology

Sample preparation	In order to isolate the stromal vascular fraction (SVF) and adipocyte fraction (AF) from eWAT and iWAT, eWAT and iWAT

	were separately minced and incubated in HEPES-buffer DMEM (Gibco, cat# 12430054) containing 10 mg/ml fatty acid-poor bovine serum albumin (BSA). After centrifuging at 500 g for 10 minutes, the pellets were incubated in the Liberase TM (0.14 units/ml, Roche, cat# 5401020001) for 30 minutes at 37 "C. The floc at the bottom was regarded as SVF and the floating cells were regarded as AF. Subsequently, the AF was digested for another 30 minutes and suspended in Dulbecco's Modified Eagle's Medium (DMEM) containing 10% FBS. The pellets and floating cells were collected after centrifuging at 500 g for 10 minutes at room temperature. These digestive steps were repeated until no precipitation occurred. The SVF was incubated in erythrocyte lysis buffer for 3 minutes at room temperature. To separate the ATMs, the erythrocyte-deleted SVF was stained and screened by flow cytometry analysis using anti-CD11b (Thermo Fisher Scientific, cat# 53-0112-80, 1:100 dilution) and anti-F4/80 (Thermo Fisher Scientific, cat# 12-4801-80, 1:100 dilution) antibodies. Bone marrow-derived macrophages (BMDMs) were detected with antibodies against F4/80 (1:80 dilution), CD11b (1:80 dilution), and perilipinl (Abcam, cat# ab3526, 1:80 dilution).
Instrument	BD FACSAria Fusion Cell Sorter
Software	BD FACSDiva software v8.0.1.
Cell population abundance	Cell populations were identified by their specific markers. At least 50000 cells were acquired for the analysis.
Gating strategy	Gating was performed using Alexa Fluor® 488, Alexa Fluor® 594, and Alexa Fluor® 405.

☒ Tick this box to confirm that a figure exemplifying the gating strategy is provided in the Supplementary Information.
